# Snijders Blok–Campeau Syndrome Associated with Pulmonary Arterial Hypertension: A Case Report

**DOI:** 10.3390/reports8020047

**Published:** 2025-04-13

**Authors:** Luisa Paul, Victoria C. Ziesenitz, Matthias Gorenflo

**Affiliations:** Pediatric Cardiology and Congenital Heart Diseases, Centre for Child and Adolescent Medicine, University Hospital, Im Neuenheimer Feld, 430, 69120 Heidelberg, Germany

**Keywords:** CHD3, Snijders Blok–Campeau syndrome, case report, vasculopathy, pulmonary arterial hypertension

## Abstract

**Background and Clinical Significance**: We report on an infant with Snijders Blok–Campeau syndrome (psychomotor developmental delay, CNS malformations) and a complex heart defect with pulmonary arterial hypertension. **Case Presentation**: A *DDX3X* mutation encoding for RNA helicase was detected, which may suggest an association between Snijders Blok–Campeau syndrome and the development of pulmonary vasculopathy. However, further validation is required. **Conclusions**: We suggest an important role for *DDX3X* in the development of the pulmonary vasculature.

## 1. Introduction and Clinical Significance

Pulmonary arterial hypertension is a serious disease that can occur in early childhood and can lead to significant morbidity and reduced life expectancy [1]. Pulmonary arterial hypertension can be associated with congenital heart disease. One of the most common genetic mutations associated with pulmonary arterial hypertension involves mutations in *BMPR2*. Rare variants are *SMAD1, SMAD4, SMAD9, CAV1, KCNK3, TBX4,* and *ACVRL1* [2].

An association between Snijders Blok–Campeau syndrome and the development of pulmonary arterial hypertension has not been characterized yet.

Snijders Blok–Campeau syndrome is a rare genetic disease usually caused by pathogenic mutations in the chromodomain helicase DNA binding protein (*CHD3*) gene. *CHD3* has a crucial role in chromatin remodeling, thereby modifying gene expressions [3]. Clinical findings include neurodevelopmental disorders (speech delay, intellectual disability, specific learning difficulties, and behavior disorders like autism spectrum disorder and attention deficit hyperactivity disorder). Facial dysmorphism, with a broad forehead, macrocephaly, hypertelorism, epicanthus, and low-set eyes, has also been previously described [4].

The Snijders Blok type of X-linked syndromic intellectual disorder is caused by a mutation in the dead-box helicase 3 gene (*DDX3X*, OMIM *300160), which encodes a protein acting as an RNA helicase and participates in a variety of cellular processes. It is, among other actors, an important regulator in the Wnt signaling pathway [5,6]. Diseases with mutations in the *DDX3X* gene can be associated with syndromic disorders with variable clinical presentation, such as varying degrees of intellectual disability, as well as developmental disorders, movement disorders, and behavioral disorders with a gender-specific effect [5]. In addition, patients show muscular hypotonia, microcephaly with potential cerebral structural abnormalities (corpus callosum hypoplasia, ventricular enlargement, cortical dysplasia), and congenital heart defects (atrial/ventricular septal defect, double orifice mitral valve, mild concentric left hypertrophy and bicuspid aortic valve) [5,7,8].

Here, we describe a female infant with a mutation in the *DDX3X* gene, pulmonary arterial hypertension, and Snijders Blok–Campeau syndrome.

## 2. Case Presentation

The parents are healthy and non-consanguineous parents. The two older siblings are healthy, with age-appropriate development. Further family history was unremarkable.

Prenatal ultrasound showed structural abnormalities of the heart, containing findings suggestive of a complex congenital heart disease (functionally univentricular heart with transposition of the great arteries). Consequently, genetic testing was initiated. A chromosome analysis from the amniotic fluid was performed in the 26th week of pregnancy and showed no numerical or structural abnormalities. Due to these findings, further genetic testing was recommended. A whole exome-trio analysis identified a heterozygous mutation (c.1600 > T; p.Arg534Cys; OMIM *300160) within the *DDX3X* gene. Neither of the parents were carriers, so a de novo mutation in the fetus was assumed.

**Figure 1:**
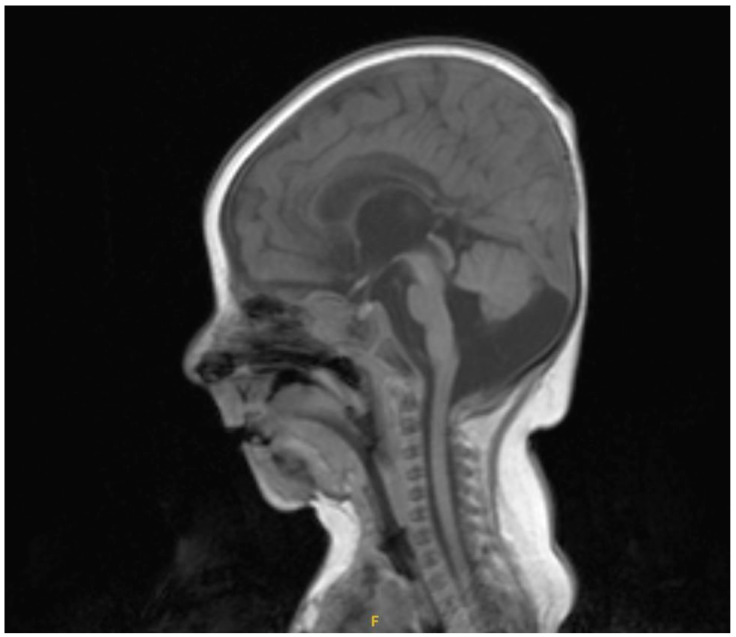
Cranial MRI image (sagittal) showing cerebral abnormalities, such as a hypoplastic cerebellar vermis with enlarged cisterna magna.

The index patient was born in gestational week 41 + 2. Birth parameters showed a low birth weight of 2190 g (−8,27z, <1st percentile). Facial abnormalities were described after birth, with retrognathia and auricular dysplasia. Prostaglandin E1 was initiated directly after birth. A complex heart defect was diagnosed by echocardiography, consisting of an univentricular heart with a dominant right ventricle, mitral valve atresia, ventricular septal defect, multifenestrated atrial septal defect, D-transposition of the great arteries, persistent left superior vena cava, patent ductus arteriosus, and coarctation of the aorta and truncus bicaroticus. Six days after birth, the first cardiac surgery with bilateral pulmonary artery banding was performed. One month later, the patient needed a cardiac catheterization with atrial septostomy , and 4 days later, a sinus-Super Flex DS stent (7 × 20 mm optimed, Ettlingen, Germany) was implanted in the patent arterial duct. A cerebral MRI was performed at the age of three months, which showed cerebral abnormalities, such as a hypoplastic cerebellar vermis with enlarged cisterna magna and a cleft palate (Figure 1).

**Figure 2:**
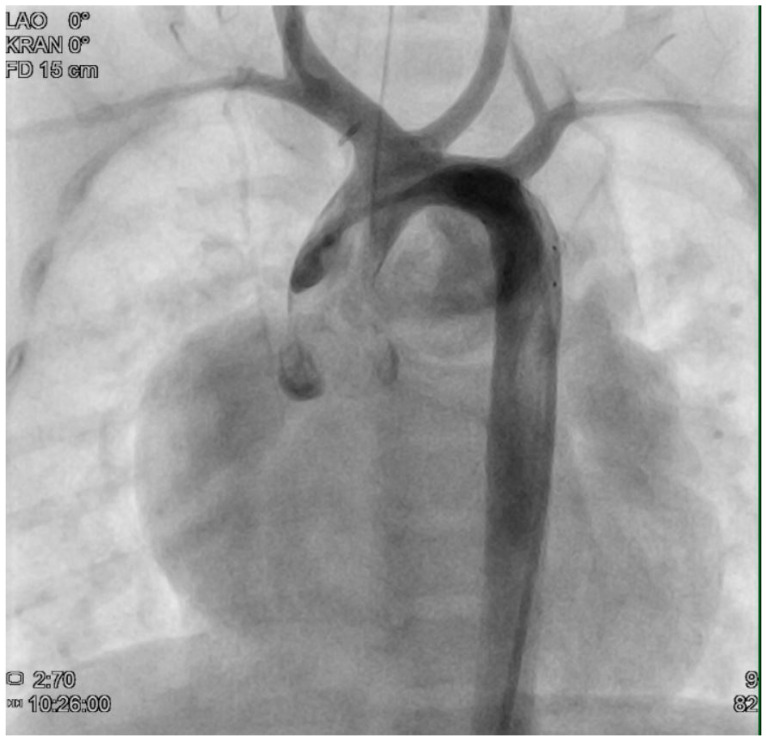
Cardiac catheterization image. Visualization of the cardiac anatomy: double outlet right ventricle, hypoplastic left ventricle, truncus bicaroticus, D-transposition of the great arteries.

**Figure 3:**
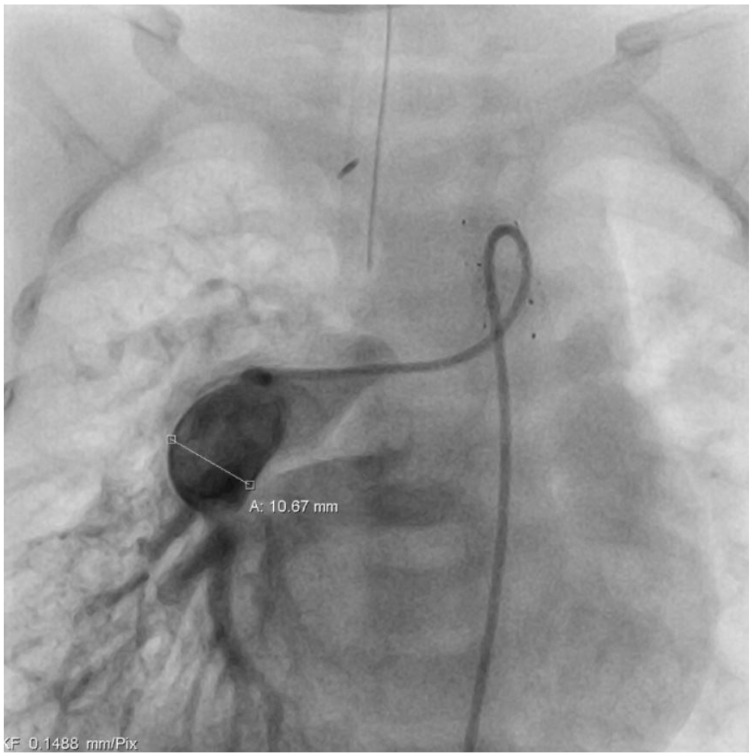
Cardiac catheterization image. The catheter is located in the RPA. There is a post-stenotic dilatation of the RPA with corkscrew-like distended peripheral pulmonary vessels as a sign of PAH.

At the age of five months, a cardiac catheter examination was performed, and severe pulmonary arterial hypertension with significantly increased pulmonary resistance was diagnosed (Table 1) despite the effective and morphologically tight bilateral pulmonary arterial banding (Figure 2 + Figure 3). Due to the pulmonary vascular pathology, a cardiosurgical correction operation does not appear to be beneficial. An off-label drug therapy with sildenafil was discussed with the parents. Despite multiple consultations, the parents refused the therapy with sildenafil because of concerns about the off-label use and side effects. Cardiac consultations take place every 3 months including echocardiography, ECG, and clinical examination.

In the long term, the disease will lead to death from right heart failure if left untreated.

## 3. Discussion

We describe a syndromic infant with a prenatally diagnosed genetic mutation in *DDX3X.* This gene defect is associated with Snijders Blok–Campeau syndrome. This syndrome is mainly associated with reduced intelligence and developmental delay. In addition to the genetic evidence, the patient has, among other features, a complex cardiac defect. Despite effective pulmonary artery banding, an invasive follow-up revealed severe pulmonary arterial hypertension.

Snijders Blok–Campeau syndrome is presumably a rare neurodevelopmental genetic disease, with only 60 cases described in the literature so far (https://medlineplus.gov/genetics/condition/snijders-blok-campeau-syndrome/#frequency accessed on 3 April 2024). It is caused by mutations in the chromodomain helicase DNA binding protein 3 on chromosome 17p13, and it is exclusively reported in female patients. All of the previously described mutations were de novo [10].

The clinical presentation of Snijders Blok–Campeau syndrome is rather broad, with intellectual disability (mild to severe), hypotonia, movement disorders, dyskinesia, spasms, a stiff-legged or wide-base gait, microcephaly, behavioral problems, and epilepsy, as well as additional features like joint hyperlaxity, skin abnormalities, cleft lip, visual impairment, and precocious puberty. MRIs often reveal cerebellar hypoplasia [5]. The previously described heart defect in our patient does not explain the secondary development of pulmonary arterial hypertension, suggesting a potential primary genetic association.

In our index patient, we found a mutation in the *DDX3X* gene, which encodes a certain kind of DEAD-box RNA helicase. Alterations of the Wnt signaling pathway have been described as contributing to pulmonary arterial hypertension [6,11].

It is important for transcription, splicing, RNA transport, and translation and has a crucial impact on cellular processes, including cell cycle control, apoptosis, and tumorigenesis. Snijders et al. proved in a knockdown *DDX3X* zebrafish a reduced brain size and microcephaly [5]. Furthermore, Mo et al. described a crucial role for *DDX3X* in tumorigenesis to metastasis [12]. In a study by Chen et al., a correlation between *DDX3X* and pulmonary fibrosis was observed. A mouse model of pulmonary fibrosis revealed enhanced survival rates and reduction in lung inflammation after administration of a *DDX3X* inhibitor [13]. You et al. revealed the hypothesis that *DDX3X* has the potential to exert an influence on vascular injury in type 2 diabetes mellitus [14]. A correlation between *DDX3X* and the development of pulmonary arterial hypertension has not been described yet. *DDX3X* is located in the cytoplasm and nucleus of the cell and has different functions depending on the localization. Rare variants such as *SMAD1*, *SMAD4*, and *SMAD9* associated with genetics with pulmonary arterial hypertension are also located in the cytoplasm and nucleus. It can therefore be assumed that there is a local relation in the mutation and that cellular processes are influenced accordingly. Presumably, there may be an association of a triggered immune reaction, which can lead to vascular remodeling and thus to pulmonary arterial hypertension [15,16].

## 4. Conclusions

Although the basic biochemical function of *DDX3X* is well understood, further research on a larger cohort of *DDX3X* patients is needed to evaluate associations with vasculopathies such as pulmonary arterial hypertension. Another possible way to evaluate *DDX3X* and PAH would be the development of an animal model (e.g., *DDX3X* mouse vs. DDX3X knockout mouse).

## Figures and Tables

**Table 1:** Hemodynamics at one month of age.

Localization	Measurements	PAH-Criteria [9]
PAPm RPA, mmHg	24	≥20 mmHg
PAPm LPA, mmHg	31	≥20 mmHg
PVRi, Wood units × m^2^	5.1	≥3 WUm²
PAWP, mmHg	12	≥10 mmHg

## Data Availability

The data presented in this study are available upon request from the corresponding author due to privacy concerns.

## Supplementary Materials

The following supporting information can be downloaded at: https://www.mdpi.com/article/10.3390/reports8020047/s1, Table S1: Literature review of relevant studies (*DDX3X*). [5,11,12,13,14].

## Abbreviations

PAPm RPA	mean pulmonary artery pressure measured in the right pulmonary artery
PAPm LPA	mean pulmonary artery pressure measured in the left pulmonary artery
PCWP	pulmonary artery wedge pressure
PVR	pulmonary vascular resistance
