# Supplementary material for: Snijders Blok–Campeau Syndrome Associated with Pulmonary Arterial Hypertension: A Case Report
Source: Reports (MDPI). 2025 Apr 13;8(2):47. doi: 10.3390/reports8020047 (PMC12196541; doi:10.3390/reports8020047)
Supplement: Supplementary file 1 [file reports-08-00047-s001.zip › reports-3516188-supplementary.pdf]

Table S1: Literature review relevant Studies (*DDX3X*)

Author	Title	Journal	Relevant findings
Singh, N. et al. (2023)	Transcriptional profiles of pulmonary artery endothelial cells in pulmonary hypertension	Nature	Alterations of the Wnt signaling pathway have been described as contributing to pulmonary arterial hypertension
Snijders Block, L. et al. (2015)	Mutations in DDX3X Are a Common Cause of Unexplained Intellectual Disability with Gender-Specific Effects on Wnt Signaling.	The American Journal of Human Genetics	A knockdown *DDX3X* zebrafish has a reduced brain size and microcephaly.
Mo, J. et al. (2021)	DDX3X: structure, physiologic functions and cancer.	Molecular Cancer	Crucial role for *DDX3X* in tumorigenesis to metastasis is assumed.
Chen, W. et al. (2023)	The mRNA-binding protein DDX3 mediates TGF-β1 upregulation of translation and promotes pulmonary fibrosis.	JCI-Insight	A correlation between *DDX3X* and pulmonary fibrosis was observed.
You, S. et al. (2023)	Down-regulation of WWP2 aggravates Type 2 diabetes mellitus-induced vascular endothelial injury through modulating ubiquitination and degradation of DDX3X.	Cardiovascular Diabetology	Hypothesis that *DDX3X* has the potential to exert an influence on vascular injury in diabetes mellitus type 2.
